# A new potential therapeutic approach for ALS: A case report with NGS analysis

**DOI:** 10.1097/MD.0000000000037401

**Published:** 2024-03-01

**Authors:** Chaur-Jong Hu, Po-Chih Chen, Neeraj Padmanabhan, Andre Zahn, Chih-Ming Ho, Kuan Wang, Yun Yen

**Affiliations:** a Department of Neurology, School of Medicine, College of Medicine, Taipei Medical University, Taipei, Taiwan; b Department of Neurology, Shuang Ho Hospital, Taipei Medical University, New Taipei City, Taiwan; c Department of Chemical and Biomolecular Engineering Henry Samueli School of Engineering at the University of California Los Angeles, Los Angeles, CA; d Department of General Medicine, Taipei Medical University Hospital, Taipei City, Taiwan; e Mechanical and Aerospace Engineering Henry Samueli School of Engineering University of California, Los Angeles, CA; f Research Center of Cancer Translational Medicine, Taipei Medical University, Taipei, Taiwan, ROC; g Graduate Institute of Cancer Biology and Drug Discovery, College of Medical Science and Technology, Taipei Medical University, Taipei, Taiwan, ROC; h Center for Cancer Translational Research, Tzu-Chi University, Hualien, Taiwan, ROC.

**Keywords:** ALS, new potential therapeutic approach, NGS

## Abstract

**Rationale:**

Amyotrophic lateral sclerosis (ALS) poses a significant clinical challenge due to its rapid progression and limited treatment options, often leading to deadly outcomes. Looking for effective therapeutic interventions is critical to improve patient outcomes in ALS.

**Patient concerns:**

The patient, a 75-year-old East Asian male, manifested an insidious onset of right-hand weakness advancing with dysarthria. Comprehensive Next-generation sequencing analysis identified variants in specific genes consistent with ALS diagnosis.

**Diagnoses:**

ALS diagnosis is based on El Escorial diagnostic criteria.

**Interventions:**

This study introduces a novel therapeutic approach using artificial intelligence phenotypic response surface (AI-PRS) technology to customize personalized drug-dose combinations for ALS. The patient underwent a series of phases of AI-PRS-assisted trials, initially incorporating a 4-drug combination of Ibudilast, Riluzole, Tamoxifen, and Ropinirole. Biomarkers and regular clinical assessments, including nerve conduction velocity, F-wave, H-reflex, electromyography, and motor unit action potential, were monitored to comprehensively evaluate treatment efficacy.

**Outcomes:**

Neurophysiological assessments supported the ALS diagnosis and revealed the co-presence of diabetic polyneuropathy. Hypotension during the trial necessitated an adaptation to a 2-drug combinational trial (ibudilast and riluzole). Disease progression assessment shifted exclusively to clinical tests of muscle strength, aligning with the patient’s well-being.

**Lessons:**

The study raises the significance of personalized therapeutic strategies in ALS by AI-PRS. It also emphasizes the adaptability of interventions based on patient-specific responses. The encountered hypotension incident highlights the importance of attentive monitoring and personalized adjustments in treatment plans. The described therapy using AI-PRS, offering personalized drug-dose combinations technology is a potential approach in treating ALS. The promising outcomes warrant further evaluation in clinical trials for searching a personalized, more effective combinational treatment for ALS patients.

## 1. Introduction

Amyotrophic lateral sclerosis (ALS) is a progressive neurodegenerative disorder characterized by anterior horn cell and corticospinal degeneration, primarily involving motor neurons in the cerebral cortex, brainstem, and spinal cord.^[1]^ Interest in research in biomarkers relevant to ALS has steadily grown over the past decade because of the recognition of their implications in therapy development. Currently, only 2 medications are US Food and Drug Administration (FDA)-approved for ALS treatment: riluzole, a glutamatergic transmitter modulator, and edaravone, an antioxidant.^[2]^ Both are limited to slowing the clinical progression of ALS by only a few months.^[3]^ Past reports have proposed various drug combinations for ALS treatment, suggesting that combinations with different mechanisms of action may be necessary in addition to the aforementioned drugs.^[4,5]^

Many other drugs have also shown neuroprotective effects, therefore, additional candidates for the treatment of ALS, in addition to the FDA-approved drugs might be warranted. Ropinirole has been suggested to possess a potential anti-ALS mechanism, including antioxidant activity, mitochondria rescue, reduction of stress granules, and modulation of abnormal proteins such as phosphorylated TAR DNA-binding protein 43 (TDP-43) and FUS RNA binding protein (FUS), as well as dopamine D2 receptor (D2R) agonism.^[6]^ Tamoxifen, an FDA-approved anticancer drug, exhibits neuroprotective effects by attenuating inflammation-mediated damage, and regulating dendritic plasticity.^[7]^ Ibudilast, a nonselective phosphodiesterase (PDE) inhibitor initially developed for pulmonary airway diseases, demonstrating anti-inflammatory and neuroprotective activities, characterized by a reduction in inflammatory cytokines, inhibition of superoxide formation, and suppression of nitrogen oxide (NO) production.^[8]^

[Link to the source: https://oce.ovid.com/article/00006024-202009030-00006]

We present a trial on the use of a 4-drug combination consisting of Riluzole, Ropinirole, and Ibudilast, and its effects on the biomarkers and clinical evaluation using AI-Phenotypic Response Surface (AI-PRS) for optimizing the individual dosing.

## 2. Case presentation

The patient, a 75-year-old East Asian male, experienced an insidious onset of weakness in his right hand 4 years ago. His hand weakness progressed to all extremities, accompanied by dysarthria, and he was later diagnosed with ALS in 2018. The weakness finally affected his respiratory muscles and he undertook tracheostomy for ventilator use. His Amyotrophic Lateral Sclerosis Functional Rating Scale (ALSFRS-R) score was 14 at admission and progression of his motor function was assessed using the Pegboard Test/hand grasp test, including muscle strength of the tongue. Plasma biomarkers related to ALS progression, including neurofilament light chain (NfL) and TDP43 were also measured every month.

The subject was hospitalized on February 27, 2020, and the clinical trial began on March 3, 2020. The trial was approved by the Institutional Review Board of Taipei Medical University (IRB number: N202002036). The trial aimed to explore optimal drug dosage combinations to maintain or improve ALS symptoms by assessment of both plasma biomarkers and clinical indicators. M doses for each drug within an N-drug combination entails navigating a vast M^N^ search space. Employing the conventional trial-and-error approach for this task proves to be impractical due to its prohibitive nature.

We utilized a small- database to train artificial neural networks, revealing that cells subjected to combinatorial drug stimulation exhibit a smooth response surface. Through regression analysis, we established the AI-PRS, represented by a polynomial-type AI-PRS function. This function characterized by (N^2^ + 3N + 2)/2 coefficients provides a quantitative relationship between a physiological response outputof a biological complex system and drug dose inputs.^[9]^ In essence, this discovery enables us to conduct only (N^2^ + 3N + 2)/2 tests instead of M^N^ searches, facilitating the identification of the globally optimal drug-dose combination for maximal efficacy. The AI-PRS platform has been validated across approximately 40 disease models, spanning in vitro,^[9]^ preclinical,^[10]^ and clinical trials.^[11]^

According to the AI-PRS approach, 15 drug-dose tests are necessary to determine a personalized optimal dose combination for a 4-drug regimen. The outcomes of the 15 drug-dose combinations may exhibit improvement or decline based on their placements on the curved AI-PRS. Once the AI-PRS is formulated, it becomes possible to identify the maximal outcome and pinpoint the optimal 4 drug-dose combinations. The initial combination, administered between March 03 and March 24, 2021, included 20 mg/d ibudilast, 25 mg/d riluzole, 20 mg/d tamoxifen, and 0.75 mg/d Ropinirole. During this period, trends in neck flexion, neck extension, right and left elbow flexion, and right and left elbow extension showed improvement. Blood tests revealed decreased levels of NfL, Creatinine Kinase (CK), and TDP-43. A second dose, administered between March 24 and April 14, 2021, comprised 40 mg/d ibudilast, 50 mg/d riluzole, 10 mg/d tamoxifen, and 0.75 mg/d Ropinirole. The patient reported a continued improvement in neck and knee extensions, along with elbow flexion. However, hypotension was observed towards the end of the second combination. A subsequent third dose, administered between April 14 and May 06, 2021, consisted of 10 mg/d Ibudilast, 12.5 mg/d riluzole, 40 mg/d tamoxifen, and 2 mg/d ropinirole. Blood tests indicated the increase of creatine kinase (CK) levels along with a decrease in neck extension muscle power. The fourth dosage, administered between May 07 and May 21, 2021, included 40 mg/d Ibudilast, 12.5 mg/d riluzole, 10 mg/d Tamoxifen, and 3 mg/d Ropinirole. Subsequent blood tests revealed decreased CK levels and increased neck extension.

After this combination, the patient reported muscle weakness, tingling, and chills, resulting in the cessation of further experimental treatment. We observed swelling in the lower limbs and persisted low blood pressure. A subsequent ultrasound examination of the lower limbs confirmed the presence of deep vein thrombosis. To address this episode, we administered 10 mg/d of rivaroxaban (Xarelto). Once the thrombus had been cleared, we resumed the experimental treatment from June 29 to July 02, 2021. We chose to administer the first 4-drug combination as the patient showed a positive response supported by both clinical assessment and biomarker levels. However, hypotension persisted throughout the treatment, leading to removing tamoxifen from the experimental regimen.

We treated the patient with a combination of ibudilast, riluzole, and ropinirole continuously. To better assess the motor function more precisely, we started the assessment with the grooved pegboard, swallowing test, and hand grip test. We administered a combination of 20 mg/d ibudilast, 25 mg/d riluzole, and 0.75 mg/d Ropinirole between July 03 and July 13, 2021. We observed a decrease in the patient’s motor function and persistent hypotension following this treatment. The patient also reported transient loss of vision and we halted the experimental treatment and removed ropinirole from the subsequent combinations.

**Figure 1.**
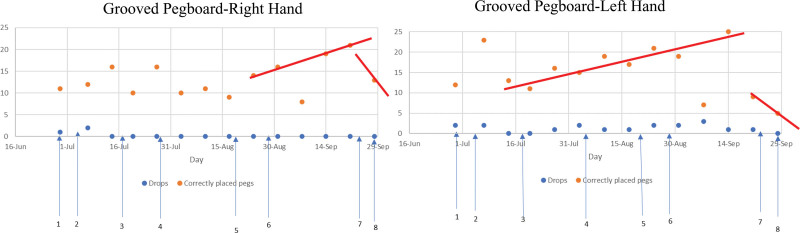
Groove pegboard test. The scores of the right-hand grooved pegboard test improved since 8/28 when regimen II-2 was given to the patient on 7/28. A clear drop happened when Regimen II-3 was given. The scores of the left-hand Grooved Pegboard Test improved since 7/13. A clear drop happened when Regimen II-3 was given.

After the patient recovered, a two-drug combination of 20 mg/d ibudilast and 25 mg/d riluzole was administered between July 28 and August 18, 2021. The patient showed improvement in the left-hand pegboard test along with increased grip strength. The patient experienced hypotension near the end of the second dose in the first week of April 202. In mid-August 2021, hypotension occurred twice a day with exact 12-hour intervals due to the use of an ophthalmic drug (Simbrinza). To eliminate hypotension, we stopped the use of brinzolamide/brimonidine tartrate (Simbrinza) and noticed that the hypotension ceased after discontinuing the use of the ophthalmic drug, brinzolamide/brimonidine tartrate (Simbrinza). Subsequently, we doubled the doses of both ibudilast and riluzole and administered another combination of 40 mg/d ibudilast and 50 mg/d riluzole between August 28 and September 26, 2021. The patient displayed a noticeable improvement in all 3 tests following this combination, as shown in Figure 1. Furthermore, we observed no incidence of hypotension.

**Figure 2.**
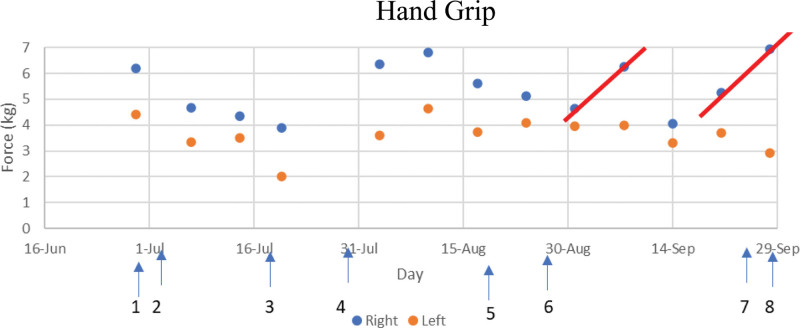
Hand grip force test. The right-hand grip tests experienced improvement since the end of August when regimen II-2 was given. The data on 9/14 was taken when the patient was sitting on the bed and usually gave low readings compared with sitting in the wheelchair. The grip force was not decreased when the patient was treated by regimen II-3. No appreciable changes have been observed for the left-hand test.

According to AI-PRS technology, only 3 dose variabions of ibudilast can identify the maximal efficacy of the two-drug combination by fixing the dose of 1 drug. We subsequently attempted to increase the ibudilast to 60 mg/d and to keep riluzole at 50 mg/d administered between September 27 and September 28, 2021. However, following this treatment, the patient experienced severely affected visual complaints, headaches, nausea, and elevated heartbeat. Additionally, a significant decrease in muscle strength was observed in the pegboard tests (Fig. 1), except for the right-hand grip (Fig. 2), leading us to halt this trial.

## 3. Discussion

The trial involved the combination of 4 drugs ibudilast, riluzole, tamoxifen, and ropinirole in the treatment of a patient with advanced ALS. To date, only riluzole and edaravone are approved for the treatment of ALS.^[4]^ These 4 drugs were meticulously selected and reviewed by team of clinicians and clinical pharmacologist. However, the optimal doses of the individual drugs are unknown. AI-PRS is a method for a rational approach to identify the best combination. The initial AI-PRS-based dose design showed increasing trends in isometric handheld dynamometer scores and decreasing trends in NfL, TDP-43, and CK. These trends were sustained until the second combination, around the first week of April 2021, when hypotension occurred. Subsequently, no clear improvement trends were observed and fifteen tests were left in complete. In contrast, the first dose yielded positive outcomes.

The current literature indicates that ibudilast alone does not sufficiently decrease glial activation in ALS patients, which is associated with ALS progression.^[12]^ However, recent studies have suggested that a combination of ibudilast and riluzole maintained or moderately improved the ALSFRS-R score in trials.^[12]^ Tamoxifen, an FDA-approved cancer drug, has been shown to limit the aggregation of TDP-43 in animal trials and has been associated with cell death in ALS patients.^[7]^

In this study, tamoxifen combined with other drugs might exert a modest effect on attenuating progression. This aligns with the results of previous trials that demonstrated a transient effect of Tamoxifen on ALS progression in patients.^[7]^ Tamoxifen was removed due to deep vein thrombosis (DVT), and concerns were raised about exacerbating DVT. Subsequent trials consisting of ibudilast, riluzole, and ropinirole revealed a decrease in the patient’s muscle strength and transient visual problems. Ropinirole was removed after visual problems. The 2 drugs, ibudilast and riluzole, at 20 mg/d ibudilast and 25 mg/d riluzole, demonstrated an improvement in muscle function, as suggested by previous literature. Further enhancement in patient muscle strength was observed when the dosages were doubled to 40 mg/d ibudilast and 50 mg/d riluzole. However, when the ibudilast dosage was increased to 60 mg/d, the patient experienced muscle weakness, decline of vision, nausea, and tachycardia.

The patient’s response to the combination of ibudilast and riluzole mirrors the results of previous studies, suggesting that a combination of riluzole and ibudilast can improve ALS progression in patients compared to single drug treatment.^[12]^ Furthermore, the patient’s response to varying dose combinations revealed that a 2-drug combination of ibudilast and riluzole conferred the most obvious improvement in the patient’s biomarkers, indicating lower dosages offering moderate improvements. Larger doses of ibudilast, however, were found to be detrimental to muscle strength.

Over the course of the study, the patient also experienced 3 major adverse events: DVT, hypotension, and vision impairment. We detected DVT at 2.5 months in the trials following the first combination of the 4-drug. Using ultrasound, we discovered that the patient had blood clots in the lower limbs that were resolved with an anticoagulant. However, the episode of DVT is not thought to be a real side effect of experimental drugs, as thrombosis is a common symptom of ALS patients with bedridden.

The patient experienced hypotension throughout the course of the trial. The current literature failed to show that any of the experimental drugs were responsible for the hypotension. Adjustments to the experimental drug combination did not have any effect on hypotension. Following observation, we determined that hypotension occurred at 12-hour intervals. A literature showed that the brinzolamide/brimonidine tartrate (Simbrinza), taken by the patient, has the potential to result in hypotension that follows circadian patterns.^[13]^ After the cessation of Simbrinza, the hypotension resolved.

The patient also reported visual problems throughout the trial. The literature shows that none of the experimental drugs are associated with vision problems. However, ophthalmologists examined the patient and suggested that vision complications might be due to the presence of cataracts. ALS is not known to affect ocular motility in most cases; however, cataract formation is common among patients with ALS. The usually off-label use of corticosteroids in patients with ALS can promote cataract formation and lead to vision loss.^[14]^ The vision loss experienced by patients is therefore unlikely to be correlated with the experimental drug combinations that we trialed.

Variants in specific genes have been recognized to be causal or associated with ALS, some of which have been extensively researched, whereas others have been recognized as having minor involvement in ALS.^[15]^ Our patient also underwent whole-genome sequencing, and we discovered polymorphisms consistent with previous literature (Table 1). The WGS FASTQ files were uploaded to the Illumina BaseSpace website. We performed sequence alignment and germline variant calling using Dragen Germline App (version 3.9.5) with the human reference genome (hg38 Alt-Aware). Variants with a PASS tag in the FILTER column of the hard-filtered VCF file were selected for variant annotation. We used ANNOVAR (2020.06.07) to identify variants that affect protein function (with GENCODE V32 gene annotation). We also utilized Qiagen HGMD Professional (2020.3) and Clinvar (20210501) to identify variants with known pathogenic information.

**Table 1:** ALS-associated gene list of study case.

Gene	Chromosome	RefID	Position start (bp)	Position end (bp)	Ref	Patient	Function	Reference
Fus	16	rs1052352	31183958	31183958	C	T	Exonic	Cantoni et al,
C9orf72	9	rs10122902	27556782	27556782	G	A	Exonic	Shatunov et al,
SETX	9	rs1056899	132264514	132264514	T	C	Exonic	Kim et al,; Shen et al,
SETX	9	rs2296871	132298298	132298298	T	C	Exonic	Kim et al,
SETX	9	rs3739922	132328143	132328143	A	C	Exonic	Shen et al,
SETX	9	rs882709	132329619	132329619	G	C	Exonic	Kim et al,
SIGMAR1	9	rs4879809	34635601	34635601	T	C	UTR3	Rheenen et al,
VAPB	20	rs1802459	58445517	58445517	A	G	UTR3	Rheenen et al,
G2E3	14	rs229195	30576390	30576390	G	A	Intronic	Rheenen et al,
MOBP	3	rs631312	39467477	39467477	G	A	Intronic	Rheenen et al,
HLA-DQB1; AL662789.1	6	rs9275477	32704864	32704864	A	C	Intergenic	Rheenen et al,
SLC9A8	20	rs17785991	49822224	49822224	T	A	Intronic	Rheenen et al,
COG3;ERICH6B	13	rs2985994	45539849	45539849	C	T	Intergenic	Rheenen et al,
ZNF512B	20	rs2275294	63962894	63962894	G	A	Intronic	Lida et al,

Of the many ALS-linked genes, the expression of variants in SOD1, TARDBP, FUS, and C9ORF72 has been the most extensively characterized.^[15]^ Variants in FUS and C9ORF72, which have been previously reported to be linked to ALS, were observed in our patient. Our patient expressed the exonic rs1052352 polymorphism of FUS, which has been previously reported to be closely linked to ALS and FTD.^[16]^ Strong evidence of polymorphisms of C9ORF72, including rs10122902, has been suggested to be genetically associated with sporadic ALS after conducting a genome-wide study and mapping gene expression.^[17]^

Besides the extensively researched ALS-linked genes mentioned above, other genes have been considered to have minor effects on the pathogenesis of ALS. The connection between a specific gene variant and the disease is not always clear due to the limited number of patients with the variant and the limited study size, giving inconclusive results. Mejzini et al^[14]^ compiled a comprehensive list of genes thought to play minor roles in ALS. Of the many genes with minor involvement mentioned, our patient observed variants in SETX, SIGMAR1, and VAPB that have been previously reported.^[14]^ Our patient expressed 4 exonic polymorphisms (rs1056899, rs2296871, rs3739922, and rs882709) that have been previously reported in SETX. SETX was also identified as a CMT-associated gene. Of these 4 variants, rs1056899, rs2296871, and rs882709 were reported by Kim et al, who observed frequencies of 0.781, 0.747, and 0.387, respectively, in Korean controls.^[18]^ Regarding SIGMAR1, the rs4879809 variant has been reported to play a role in the pathogenesis of ALS in a Pakistani family.^[19]^ VAPB is another gene considered to be a gene with minor involvement in ALS. The rs1802459 variant has been suggested to play a pathogenic role in familial ALS.^[20]^ Our patient also expressed polymorphisms in G2E2, MOBP, HLA-DOB1, SLC9A8, and COG3 as reported by Rheenen et al^[21]^ as genome-wide significant loci.

Our patient expressed the rs2275294 variant of the ZNF512B gene. This specific variant was first reported as having an increased risk of ALS in the Japanese population after performing SNP analysis using the GenBank of Japanese ALS patients^[22]^ and suggested that the rs2275294 variant could also be a new prognostic factor in ALS.^[23]^ Meta-analyses results showed that the rs2275294 variant increased the risk of ALS, especially in Asian populations.^[24,25]^

Finally, several limitations must be acknowledged to provide a comprehensive interpretation of our findings. One of the major limitations of our study is single patient study. Although the personalized approach produced intriguing results, the applicability of these findings to a broader ALS population may be restricted. Future studies with larger cohorts are necessary to validate and extend our observations. Second, adverse events, including hypotension, deep vein thrombosis, and vision impairment, were encountered during the trial. These events significantly influenced the trial’s course and may impact the broader applicability of the proposed drug combinations. Third, discontinuation of Tamoxifen, the removal of tamoxifen from the drug combination due to concerns about deep vein thrombosis limited the exploration of its potential synergistic effects with other drugs. This discontinuation highlights the challenges and potential risks associated with combining multiple medications in ALS treatment. Fourth, the genetic analysis identified variants in specific ALS-linked genes and genetic variability should be involved in the diagnosis and response to treatment. However, our study did not explore the functional impact of these variants. Further investigations are essential to establish fundamental relationships between these genetic factors and treatment outcomes. Despite these limitations, our study contributes valuable insights into the potential of personalized drug combinations in ALS treatment. Interpretation of these findings should consider the outlined constraints, and future research efforts need to tackle these limitations to achieve a more comprehensive understanding of the therapeutic landscape in ALS.

## 4. Conclusion

Based on the present results, we suggest that using AI-PRS technology is a potential approach to treating ALS with personalized optimal drug-dose combinations and warrants further evaluation in clinical trials.

## Author contributions

**Conceptualization:** Yun Yen, Chih-Ming Ho.

**Data curation:** Chaur-Jong Hu, Po-Chih Chen, Neeraj Padmanabhan.

**Formal analysis:** Neeraj Padmanabhan.

**Funding acquisition:** Yun Yen.

**Investigation:** Yun Yen.

**Methodology:** Po-Chih Chen, Andre Zahn.

**Project administration:** Chaur-Jong Hu.

**Supervision:** Yun Yen, Chih-Ming Ho, Kuan Wang.

**Validation:** Chaur-Jong Hu.

**Writing – original draft:** Neeraj Padmanabhan, Andre Zahn.

**Writing – review & editing:** Yun Yen, Chih-Ming Ho, Kuan Wang.

## References

[1] Brown RH Al-Chalabi A. Amyotrophic lateral sclerosis. N Engl J Med. 2017;377:162–72. 28700839 10.1056/NEJMra1603471

[2] Bensimon G Lacomblez L Meininger V. A controlled trial of riluzole in amyotrophic lateral sclerosis. ALS/Riluzole Study Group. N Engl J Med. 1994;330:585–91. 8302340 10.1056/NEJM199403033300901

[3] Writing Group; Edaravone (MCI-186) ALS 19 Study Group. Safety and efficacy of edaravone in well defined patients with amyotrophic lateral sclerosis: a randomised, double-blind, placebo-controlled trial. Lancet Neurol. 2017;16:505–12. 28522181 10.1016/S1474-4422(17)30115-1

[4] Kriz J Gowing G Julien JP. Efficient three-drug cocktail for disease induced by mutant superoxide dismutase. Ann Neurol. 2003;53:429–36. 12666110 10.1002/ana.10500

[5] Waibel S Reuter A Malessa S. Rasagiline alone and in combination with riluzole prolongs survival in an ALS mouse model. J Neurol. 2004;251:1080–4. 15372249 10.1007/s00415-004-0481-5

[6] Okano H Yasuda D Fujimori K. Ropinirole, a new ALS drug candidate developed using iPSCs. Trends Pharmacol Sci. 2020;41:99–109. 31926602 10.1016/j.tips.2019.12.002

[7] Chen PC Hsieh YC Huang CC. Tamoxifen for amyotrophic lateral sclerosis: a randomized double-blind clinical trial. Medicine (Baltimore). 2020;99:e20423. 32481440 10.1097/MD.0000000000020423 PMC12245230

[8] Bhat A Ray B Mahalakshmi AM. Phosphodiesterase-4 enzyme as a therapeutic target in neurological disorders. Pharmacol Res. 2020;160:105078. 32673703 10.1016/j.phrs.2020.105078

[9] Al-Shyoukh I Yu F Feng J. Systematic quantitative characterization of cellular responses induced by multiple signals. BMC Syst Biol. 2011;5:88. 21624115 10.1186/1752-0509-5-88 PMC3138445

[10] Lee BY Clemens DL Slive A. Drug regimens identified and optimized by output-driven platform markedly reduce tuberculosis treatment time. Nat Commun. 2017;8:14183. 28117835 10.1038/ncomms14183 PMC5287291

[11] Zarrinpar A Lee DK Silva A. Individualizing liver transplant immunosuppression using a phenotypic personalized medicine platform. Sci Transl Med. 2016;8:333ra–49. 10.1126/scitranslmed.aac5954 27053773

[12] Oskarsson B Maragakis N Bedlack RS. MN-166 (ibudilast) in amyotrophic lateral sclerosis in a Phase IIb/III study: COMBAT-ALS study design. Neurodegener Dis Manag. 2021;11:431–43. 34816762 10.2217/nmt-2021-0042

[13] Seibold LK DeWitt PE Kroehl ME. The 24-hour effects of brinzolamide/brimonidine fixed combination and timolol on intraocular pressure and ocular perfusion pressure. J Ocul Pharmacol Ther. 2017;33:161–9. 28129020 10.1089/jop.2016.0141 PMC5385426

[14] Cenk Kohen M Beril Kucumen R. Cataract surgery in a patient with amyotrophic lateral sclerosis: a case report. Case Rep Ophthalmol. 2011;2:198–204. 21886620 10.1159/000329832 PMC3130492

[15] Mejzini R Flynn LL Pitout IL. ALS genetics, mechanisms, and therapeutics: where are we now? Front Neurosci. 2019;13:1310. 31866818 10.3389/fnins.2019.01310 PMC6909825

[16] Cantoni C Fenoglio C Cortini F. FUS/TLS genetic variability in sporadic frontotemporal lobar degeneration. J Alzheimers Dis. 2010;19:1317–22. 20061612 10.3233/JAD-2010-1328

[17] Shatunov A Mok K Newhouse S. Chromosome 9p21 in sporadic amyotrophic lateral sclerosis in the UK and seven other countries: a genome-wide association study. Lancet Neurol. 2010;9:986–94. 20801717 10.1016/S1474-4422(10)70197-6 PMC3257853

[18] Kim HJ Hong YB Park JM. Mutations in the PLEKHG5 gene is relevant with autosomal recessive intermediate Charcot-Marie-Tooth disease. Orphanet J Rare Dis. 2013;8:104. 23844677 10.1186/1750-1172-8-104 PMC3728151

[19] Ullah MI Ahmad A Raza SI. In silico analysis of SIGMAR1 variant (rs4879809) segregating in a consanguineous Pakistani family showing amyotrophic lateral sclerosis without frontotemporal lobar dementia. Neurogenetics. 2015;16:299–306. 26205306 10.1007/s10048-015-0453-1

[20] Chen HJ Anagnostou G Chai A. Characterization of the properties of a novel mutation in VAPB in familial amyotrophic lateral sclerosis. J Biol Chem. 2010;285:40266–81. 20940299 10.1074/jbc.M110.161398 PMC3001007

[21] van Rheenen W van der Spek RAA Bakker MK.; SLALOM Consortium. Common and rare variant association analyses in amyotrophic lateral sclerosis identify 15 risk loci with distinct genetic architectures and neuron-specific biology. Nat Genet. 2021;53:1636–48. 34873335 10.1038/s41588-021-00973-1 PMC8648564

[22] Iida A Takahashi A Kubo M. A functional variant in ZNF512B is associated with susceptibility to amyotrophic lateral sclerosis in Japanese. Hum Mol Genet. 2011;20:3684–92. 21665992 10.1093/hmg/ddr268

[23] Tetsuka S Morita M Iida A. ZNF512B gene is a prognostic factor in patients with amyotrophic lateral sclerosis. J Neurol Sci. 2013;324:163–6. 23168171 10.1016/j.jns.2012.10.029

[24] Ning P Yang X Yang B. Meta-analysis of the association between ZNF512B polymorphism rs2275294 and risk of amyotrophic lateral sclerosis. Neurol Sci. 2018;39:1261–6. 29713939 10.1007/s10072-018-3411-5

[25] Jiang H Yang B Wang F. Association of single nucleotide polymorphism at rs2275294 in the ZNF512B gene with prognosis in amyotrophic lateral sclerosis. Neuromolecular Med. 2021;23:242–6. 33387304 10.1007/s12017-020-08634-y

